# Interrelation Between BMI, Dietary Habits, Self-Rated Health, and Body Image Perception Among Korean Adolescents: The Korea Youth Risk Behavior Web-Based Survey (2022)

**DOI:** 10.3390/nu17020253

**Published:** 2025-01-11

**Authors:** Jeongha Oh, Woo-Lim Mun, Ye-Eun Lee, Su-Yeon Roh, Geunkook Kim

**Affiliations:** 1 Department of Dance, Sejong University, Seoul 05006, Republic of Korea; thebalance01@naver.com; 2 Department of Exercise Rehabilitation, Gachon University, Incheon 21936, Republic of Korea; woolim@gachon.ac.kr; 3 Department of Health Science, Gachon University Graduate School, Incheon 21936, Republic of Korea; 4 Department of Sports Rehabilitation, Jaeneung University, Incheon 22573, Republic of Korea

**Keywords:** adolescent, BMI, dietary habits, self-rated health, body image perception

## Abstract

Background/Objectives: Adolescent obesity is highly likely to lead to adult obesity and is associated with dietary habits, subjective health, and body image perception. This study aimed to analyze the relationships between BMI, dietary habits, subjective health perception, and body image perception among Korean adolescents using data from the 18th Korea Youth Risk Behavior Survey conducted in 2022 to explore strategies for reducing adolescent obesity rates. Methods: Data from 50,427 participants were analyzed, including BMI, seven lifestyle factors (intake frequencies of water, milk, fruit, soft drinks, vegetables, breakfast, and late-night snacks), and responses to one item each for subjective health perception and body image perception. Results: Higher intake frequencies of breakfast, fruits, soft drinks, and late-night snacks were associated with lower BMI (*p* < 0.001). However, among high school students, those with lower water and breakfast intake but higher soft drink and late-night snack intake exhibited an increasing trend in BMI. Subjective health perception and body image perception were interrelated, with subjective health perception influencing body image perception (*p* < 0.001). Conclusions: Addressing nutritional issues within schools, including improving school meals, regulating accessible products, and providing nutritional intake guidelines, is essential. Additionally, developing tailored health education programs to promote healthy body image perceptions is necessary. This study can serve as a foundational resource for analyzing adolescent health and developing strategies to improve health behaviors in changing environments.

## 1. Introduction

Obesity among Korean adolescents has recently gained significant social attention, owing to its associated health problems. According to the 2021 Student Health Examination Sample Statistics Analysis, published by the Ministry of Education, the obesity rate among adolescents increased significantly from 15.1% in 2019 to 19% in 2021, marking a substantial rise of 3.9%, which highlights the need to strengthen the national obesity management system [1]. Furthermore, the World Obesity Atlas 2023, published by the World Obesity Federation (WOF), predicts a rapid increase in global obesity rates, including among adolescents, by 2035 [2,3].

Obesity has only recently been recognized as a social problem in Korea. In the 2000s, concerns about adolescent obesity in Korea began to surface, and in 2005, the Korea Youth Policy Institute raised alarms over the rapid increase in obesity rates among Korean adolescents, drawing growing attention to the issue. Since then, the obesity rate among Korean adolescents has steadily increased annually; within just two decades, it has become one of the most serious social issues threatening adolescent health.

Common causes of obesity among adolescents include skipping breakfast, an imbalance in fruit and vegetable consumption, and an increased intake of fast food and convenience meals [4,5]. This has been attributed to the higher exposure of Korean adolescents to high-calorie foods such as fast food, processed foods, and sugary drinks compared to the average in the World Health Organization (WHO) East Asia region, a finding supported by previous research [5]. The increasing demand for high-calorie foods has led to a higher density of fast-food restaurants, thereby increasing the accessibility of convenience foods and creating a vicious cycle [6,7]. Furthermore, the COVID-19 pandemic, which drastically altered lifestyles worldwide, has accelerated the surge in obesity rates. Students who lost access to balanced school meals and regular exercise opportunities naturally experienced physical inactivity and nutritional imbalances, leading to increased obesity rates, a trend already reported in numerous studies [8,9,10,11].

The negative impact of obesity on adolescent health is widespread. Obesity affects not only the physical, mental, and emotional health of adolescents but also their perception of their body and overall health. In addition, the rising healthcare costs associated with preventing and treating obesity have emerged as a significant social issue, warranting national policy-level attention and intervention [12]. Adolescent obesity increases the risk of various physical health problems, including cardiovascular diseases, type 2 diabetes, hypertension, and sleep apnea [13]. Obesity that begins in childhood often persists into adulthood [14], potentially exacerbating health problems later in life. Moreover, adolescent obesity is significantly associated with an increased likelihood of mental health issues such as depression, anxiety, and low self-esteem [15]. In particular, negative perceptions of body image may develop, leading to negative feedback or bullying from peers, which can have a detrimental psychological impact. Negative experiences, such as stigma or discrimination, can undermine self-efficacy and contribute to long-term body image distortion. This may obscure understanding of the relationship between obesity and health, diminish awareness of related complications, and alter subjective health perceptions [16]. Therefore, it is crucial to address these issues through dedicated attention and intervention [17,18]. Accordingly, it is essential not only to consider measures to reduce the rising obesity rates among adolescents but also to comprehensively address their physical and mental health. Efforts should encompass enhancing adolescents’ health awareness, adopting a comprehensive approach to their overall well-being, and developing holistic strategies to address the issue effectively.

Previous studies have attempted to identify factors associated with obesity (or BMI) in adolescents by analyzing physical abilities such as cardiorespiratory fitness [19,20] or metabolic systems [21]. In addition, some dietary factors (eating speed, nutrient intake ratio) have been studied in adolescents to determine the impact of dietary habits on obesity and BMI [22,23]. However, most studies have been conducted with small sample sizes, which limits the ability to identify overall trends.

Therefore, this study aims to analyze the relationships between BMI, dietary habits, subjective health, and body image among Korean adolescents through a comprehensive large-scale survey. This aims to identify factors that threaten the physical and mental health of adolescents through various dietary habit factors and a large sample size. Based on the results of this study, we hope to raise awareness of the relationship between dietary habits and BMI, physical and psychological factors, and ultimately propose strategies to improve the overall health and well-being of adolescents through accurate information and standards.

## 2. Materials and Methods

### 2.1. Sample

The participants of this study were adolescents enrolled in middle and high schools across South Korea who participated in the 18th Youth Health Behavior Survey jointly conducted by the Korea Disease Control and Prevention Agency and the Ministry of Education in 2022. The survey targeted students from 7th grade (1st year of middle school) to 12th grade (3rd year of high school) and was conducted as a government-approved statistical survey (Approval No. 117058). In total, 51,850 adolescents responded to the survey. However, after excluding cases with nonresponse and missing data, 50,427 participants were included in the final analysis.

### 2.2. Measures

Data were collected through an anonymous self-administered online survey. The survey was conducted in computer labs with Internet access, where each participant was randomly assigned to an individual computer. In schools with limited access to computer labs, the survey was conducted using tablet PCs or smartphones. Descriptions of the survey variables used in this study are as follows.

#### 2.2.1. Body Mass Index (BMI)

The BMI (kg/cm^2^) was calculated from self-reported height and weight, rather than direct anthropometric measurements. Self-reported BMI calculations can lead to an over- or underestimation of results [24] but are known to be useful for identifying time series trends in large samples [25]. In this study, we also attempted to analyze BMI < 18.5 as underweight, 18.5 ≤ BMI ≤ 23.0 as normal, and BMI > 23.0 as overweight [26].

#### 2.2.2. Dietary Habits

The dietary habits questionnaire comprised seven items. This questionnaire was developed based on food-based dietary recommendations for Korean adolescents [27]. One of the items assessed was water intake, with the question (i) “In the past 7 days, how often did you drink water each day?”. Responses were categorized as “Less than 1 cup per day”, “1–2 cups per day”, “3 cups per day”, “4 cups per day”, and “5 or more cups per day”. (ii) Breakfast intake was assessed with the question “In the past 7 days, on how many days did you eat breakfast (excluding only milk or juice)?”. Responses ranged from “0 days” to “7 days”. (iii) Milk intake was assessed with the question “In the past 7 days, how often did you drink milk?”. (iv) Soft drink intake was assessed with the question “In the past 7 days, how often did you drink sweetened beverages?”. (v) Late-night snack intake was assessed with the question “In the past 7 days, how often did you eat late-night snacks?”. (vi) Fruit intake was assessed with the question “In the past 7 days, how often did you eat fruits (excluding fruit juice)?”. (vii) Vegetable intake was assessed with the question “In the past 7 days, how often did you eat vegetables (excluding kimchi) during meals?”. Responses were categorized as “Did not eat in the past 7 days”, “1–2 times per week”, “3–4 times per week”, “5–6 times per week”, and “7 or more times per week”.

#### 2.2.3. Self-Rated Health

The self-rated health item asked “How would you describe your overall health status?”. Responses were categorized as follows: “1 = Very healthy”, “2 = Healthy”, “3 = Average”, “4 = Unhealthy”, and “5 = Very unhealthy”.

#### 2.2.4. Body Image Perception

The body image perception item asked “How would you describe your body type?”. Responses were categorized as follows: “1 = Very thin”, “2 = Slightly thin”, “3 = Average”, “4 = Slightly overweight”, and “5 = Very overweight”. In previous studies, body image perception responses were categorized into thin, normal, and overweight [28], but in this study, the responses to thin and overweight were further categorized into ‘slightly’ and ‘very’.

### 2.3. Statistical Analysis

Statistical analysis was performed using SPSS software (version 28.0; IBM Corp., Armonk, NY, USA). Frequency analysis was conducted to examine the response distributions for general characteristics, dietary habits, self-rated health, and body image perception. An independent t-test was used to examine differences in BMI, dietary habits, self-rated health, and body image perception based on sex. One-way ANOVA was used to analyze differences in BMI, dietary habits, self-rated health, and body image perception based on school type and grade, differences in BMI based on dietary habits, and differences in self-rated health and body image perception based on BMI. We controlled for confounding variables (e.g., gender, school type, grade level) that could affect the results. All statistical significance levels were set at 5%.

## 3. Results

Table 1 presents the response distribution of study participants’ general characteristics, BMI, dietary habits, self-rated health, and body image perception. Among the participants (*n* = 50,427), 51.0% were male (*n* = 25,731) and 49.0% were female (*n* = 24,696).

The most common school type was mixed schools (68.5%, *n* = 34,511), followed by boys’ schools (15.8%, *n* = 7963) and girls’ schools (15.7%, *n* = 7913). Regarding grade distribution, 17.9% were in 7th grade (*n* = 9027), 18.0% in 8th grade (*n* = 9094), 18.2% in 9th grade (*n* = 9168), 16.3% in 10th grade (*n* = 8231), 15.4% in 11th grade (*n* = 7765), and 14.2% in 12th grade (*n* = 7142). Regarding BMI distribution, 23.7% were categorized as underweight (*n* = 11,958), 47.1% as normal weight (*n* = 23,773), and 29.1% as overweight (*n* = 14,696). For daily water intake, the most common response was five or more cups (36.8%, *n* = 18,554), while the least common was less than one cup (4.3%, *n* = 2181). For weekly milk intake, the most common response was 1–2 times (29.1%, *n* = 14,683), whereas the least common was 3–5 times (11.8%, *n* = 5970). Regarding weekly soft drink intake, the most common response was 3–4 times (34.2%, *n* = 17,222), whereas the least common was 0 times (6.5%, *n* = 3297). For weekly breakfast intake, the most common response was 7 days (26.8%, *n* = 13,526), whereas the least common was 3–4 days (17.0%, *n* = 7440). For weekly late-night snack intake, the most common response was zero days (40.4%, *n* = 20,352), whereas the least common was seven days (2.0%, *n* = 998). For weekly fruit intake, the most common response was 1–2 times (32.2%, *n* = 16,244), whereas the least common was 5–6 times (10.6%, *n* = 5362). Regarding weekly vegetable intake, the most common response was seven or more times (34.8%, *n* = 17,542), whereas the least common was zero times (4.2%, *n* = 2132). For self-rated health, the most common response was healthy (43.4%, *n* = 21,903), whereas the least common was very unhealthy (0.6%, *n* = 315). For body image perception, the most common response was normality (36.5%, *n* = 18,397), whereas the least common was very thin (4.9%, *n* = 2485).

Table 2 presents the differences in BMI, dietary habits, subjective self-rated health, and body image perception according to sex, school type, and grade among the study participants. Regarding sex differences, males had higher values than females in BMI, water intake, milk intake, soft drink intake, breakfast intake, late-night snack intake, and vegetable intake. However, females scored higher than males in self-rated health and body image perception. There was no significant difference in fruit intake. For school type, boys’ schools had the highest values for BMI, water intake, milk intake, soft drink intake, and vegetable intake, whereas girls’ schools had the lowest values. For late-night snack intake, boys’ schools had the highest value. For fruit intake, mixed schools scored higher than girls’ schools. For self-rated health, the order was girls’ schools, mixed schools, and boys’ schools. For body image perception, the order was girls’ schools, boys’ schools, and mixed schools. BMI increased progressively with grade level. Water intake was lower in grades 10–12 than in other grades. Milk intake was in the order of 7–8th, 9th, 10–11th, and 12th grades. Soft drink intake was higher in grades 10–12 than in other grades. Breakfast intake was lower in grades 11–12 than in the other grades, but higher in grades 7–8. Late-night snack intake was highest in 12th grade; fruit intake was highest in grades 7–8, while vegetable intake was highest in grades 7–9. Self-rated health was higher in grades 10–12, and body image perception was highest in grade 12.

Table 3 presents the differences in BMI based on the dietary habits of the participants. As the frequency of water intake increased, BMI also tended to increase. For milk intake, BMI was higher in the group consuming milk 3 or more times per week compared to the group consuming milk 0–2 times. For soft drink intake, BMI was lower in the group consuming soft drinks seven or more times per week compared to other frequency groups. For breakfast intake, BMI was highest in the 0–4 days group, followed by the 5–6 days and 7 days groups. For late-night snack intake, BMI was lowest in the 5–7 days group, and BMI increased as the frequency of late-night snacks decreased. For fruit intake, BMI was highest in the 0–2 times group, and it decreased progressively as the frequency increased. For vegetable intake, BMI was higher in the 3 or more times group compared to the 0–2 times group. Additionally, BMI was higher in the 3–4 times group than in the 5–6 times group.

Table 4 presents the differences in self-rated health and body type according to BMI categories (underweight, normal, and overweight). Adolescents with normal BMI reported significantly healthier self-rated health than those categorized as underweight or overweight. For body image perception, adolescents with a lower BMI were more likely to perceive themselves as thin.

## 4. Discussion

This study aims to analyze the relationships between BMI, dietary habits, subjective health, and body image perception among Korean adolescents, with the goal of exploring strategies to promote adolescent health.

The most noteworthy finding based on general characteristics is the severity of obesity among Korean adolescents. According to the findings of this study, the obesity rate among Korean adolescents, as revealed in the 18th Korea Youth Risk Behavior Survey conducted in 2022, was 29.1%. This represents a significant increase of 10.1% compared to the 19% reported in the 2021 Student Health Examination [1]. Although BMI is not a direct measure of body fat and relies solely on height and weight to indicate obesity, raising concerns about its accuracy in cases of high muscle mass or unusual fat distribution [29], it is widely used as an initial screening tool for obesity in medical settings and large-scale population surveys, demonstrating considerable reliability [30]. Therefore, the steadily increasing obesity rates since 2019 warrant significant attention. The obesity rate among Korean adolescents is already significantly higher than the East Asian regional average as defined by the WHO [31]. Furthermore, compared to research findings indicating that approximately 20% of adolescents worldwide were overweight or obese as of 2022 [32], the obesity rate among Korean adolescents is notably higher. This highlights the exceptional and alarming nature of this issue, warranting urgent recognition and action. The period during which obesity rates among Korean adolescents sharply increased coincided with the global outbreak of the COVID-19 pandemic, which brought significant changes to daily life.

The surge in obesity rates during this period became a critical global health concern. Social isolation and restrictions on daily activities led to reduced physical activity, while irregular mealtimes and eating habits became prevalent. Additionally, heightened stress and anxiety were identified as contributing factors that drove individuals to overeat and opt for highly palatable, unhealthy foods [33,34,35]. A lack of access to balanced school meals and reduced physical activity directly contributed to the rise in obesity rates. Additionally, heightened academic competition, increased academic stress, changes in educational and examination formats, and the anxiety caused by sudden environmental shifts during the pandemic further exacerbated stress levels and sleep deprivation, both of which served as major contributors to obesity [36,37]. Moreover, increased use of digital devices among Korean students has been identified as a major contributing factor to obesity. During the COVID-19 pandemic, the widespread adoption of online learning significantly increased digital device usage among Korean students [38,39], which has since become a routine part of their daily lives. South Korea ranks among the top countries in terms of digital device usage and digital infrastructure [40,41]. With a smartphone penetration rate of approximately 95%, one of the highest in the world, the country also boasts rapid internet speeds and extensive internet accessibility, further facilitating the widespread use of digital devices [40,41,42,43]. Increased screen time associated with digital device use among Korean adolescents with low physical activity levels has been identified as a factor that prolongs sedentary behavior, thereby increasing the risk of obesity. Adolescent obesity is associated with musculoskeletal problems, sleep apnea, and an increased risk of early-onset cardiovascular disease. It also elevates the risk of developing chronic diseases in adulthood. Furthermore, obese adolescents are more likely to experience mental health issues, such as depression and low self-esteem, than their non-obese peers [44]. Health issues related to obesity among Korean adolescents are becoming more critical than ever before. Although their daily life has returned to normal, adolescents are expected to take considerable time to restore their disrupted dietary and lifestyle habits. Therefore, it is essential to identify additional contributing factors and address the issue of collective attention and intervention at the national level.

Next, the obesity rates based on the personal characteristics of the participants revealed that BMI increased as grade level increased. Compared to middle school students, high school students had a lower frequency of water intake, a higher frequency of soft drink consumption, less frequent breakfast consumption, and more frequent late-night snacking. These trends were particularly pronounced among 12th-grade students. Additionally, the frequency of fruit and vegetable consumption was lower among high school students than among middle school students. High school students also had a higher proportion of negative responses regarding their perceived health status and their body image was perceived to be close to being overweight. These findings suggest that high school students, particularly 12th graders, face significant challenges in maintaining healthy dietary habits. This is likely attributable to the increase in time spent at school and private academies due to academic and entrance exam pressures as students advance to higher grade levels, resulting in a significant reduction in physical activity [45]. Despite awareness of the importance of breakfast, fresh fruits, and vegetables, as well as issues with sweetened beverages and late-night snacks, the reality of students’ daily routines makes it challenging to maintain healthy dietary habits. Therefore, it is essential to address and improve nutritional practices in schools where students spend most of their time. It is crucial to strengthen nutrition education within schools and promote healthy eating habits. This effort should include not only providing healthy and nutritious school meals but also offering wholesome snack options and improving the quality of food items sold in school stores. Collaboration among schools is essential to implement these measures effectively [46,47].

Additionally, the differences in self-rated health and body image perception based on sex are noteworthy. Although male students had higher BMI levels than female students, female students reported higher scores on self-rated health and body image perception. Female students tend to perceive their body image more overweight than their actual body shape [38,39,40,41,42,43,44,45,46,47,48,49]. This is likely closely related to societal beauty standards. This suggests that subjective perspectives on health, appearance, and body shape are more influential than BMI values. Factors such as excessive emphasis on appearance propagated through the media, societal expectations of femininity, and cultural attitudes are expected to play significant roles in shaping these perceptions [48,50]. When female students are frequently subjected to overweight evaluations for various reasons, dissatisfaction with their body image may lead to mental health issues. Therefore, it is crucial to help individuals develop an objective and healthy perspective of their bodies, emphasizing the necessity of education and counseling to enhance self-esteem and promote a positive body image [51]. Furthermore, media literacy education, which cultivates the ability to critically and actively analyze various media content, has been shown to help students recognize realistic beauty standards [52]. These findings suggest that it is necessary to adopt a receptive approach to diverse educational methods.

Let us now consider the differences in BMI based on dietary habits. Higher breakfast frequency and greater fruit consumption were associated with lower BMI. Several previous studies have demonstrated the beneficial effects of breakfast on weight management and overall health [53,54,55,56]. Furthermore, the consumption of fresh fruits has been shown to yield significant results not only for weight management but also for height growth [57,58]. However, the divergence from existing research findings regarding other items is particularly noteworthy in this study. Higher consumption of soft drinks, and late-night snacks was associated with a lower BMI, contrary to the results reported in previous studies. According to previous studies, the consumption of sugar-sweetened beverages contributes to weight gain and obesity. Research on the relationship between beverage consumption and obesity has primarily focused on sugar-sweetened beverages [59,60]. In contrast, studies on water consumption suggest that groups with higher water intake have lower BMI, body fat percentage, and abdominal fat than groups with lower water intake. Thus, adequate water consumption aids weight management [61,62]. Additionally, the correlation between late-night snacking and BMI has been well documented in many studies, indicating that frequent late-night snacking significantly affects BMI [63,64]. This aligns with the findings of this study; twelfth-grade students with the highest frequency of late-night snacking also exhibited the highest BMI levels. However, the findings of this study significantly contradict conventional knowledge. It is worth noting that some studies also report outcomes that diverge from conventional understanding, similar to the results observed here. These studies provide important insights as they reflect the complex interplay of various factors influencing obesity, such as the characteristics of the study population, differences in intake levels, and other lifestyle habits. First, some studies found that the relationship between sugar-sweetened beverage consumption and obesity is unclear. According to these findings, when beverage consumption is low, the association with weight gain and obesity tends to be weak or nonexistent [65]. Additionally, other studies have concluded that the relationship between water intake and weight management is relatively weak [66]. Researchers have noted that the mechanism by which water intake affects weight is not clearly understood and that when considering individual dietary and lifestyle habits, the effect of water consumption is relatively minor. Lastly, some studies have argued that late-night snacking is not directly associated with weight gain [50]. These studies suggest that the type of food and frequency of late-night snacking play a more significant role, and in some cases, individuals who consume late-night snacks may not experience any impact on obesity. In other words, the conclusion was drawn that consuming high-quality foods as late-night snacks has little impact on weight gain. Furthermore, while late-night snacking may be related to weight gain, researchers have argued that overall caloric intake and daily meal patterns play a more significant role in influencing weight. The conclusion is that consuming low-calorie foods as late-night snacks may not significantly affect weight gain [50,67]. These contrasting findings highlight various factors that influence obesity. The relationship between late-night snacking and obesity also varies depending on the type of food and calorie intake, highlighting the need to reevaluate the association between BMI and dietary habits. To maintain a healthy weight, it is essential to emphasize that balanced eating and regular physical activity are more important than merely eating more or less food. Furthermore, additional research based on reliable data is necessary to gain deeper insight into these relationships.

Finally, subjective health and body image perception based on BMI levels revealed that adolescents with normal BMI were more likely to respond positively about their health. Adolescents with lower BMI levels perceived themselves to be thin. Previous studies on subjective health have also shown that those within the normal BMI range tend to perceive their health more positively, while the proportion of individuals perceiving themselves as healthy decreases among those with obesity [50,51]. The findings of this study also support those of previous research, suggesting that adolescents have a relatively accurate perception of their current health status. However, subjective body image perception has been a source of concern because of increasing body image distortion among adolescents. Male students tend to perceive their bodies as smaller or thinner than they actually are, while female students are more likely to evaluate themselves as overweight [67,68]. This body image distortion phenomenon is presumed to stem from idealized body standards promoted by mass media—muscular and robust physiques for males and slim, slender bodies for females—leading individuals to compare themselves and distort their own body image. Compared to other countries, including the United States, the level of distortion among adolescents in Korea has been notably severe, prompting numerous studies on the problems associated with body image distortion and potential solutions [69,70]. Although this study did not analyze the proportions of body image perception according to BMI categories, it found that adolescents with a normal BMI tended to perceive their health positively, whereas those with a lower BMI were more likely to perceive their body as thinner. Additionally, the findings indicate that better subjective health status is associated with a more positive body image perception. Based on these findings, it is necessary to develop tailored health education programs, that consider differences in body image perception, to foster a healthy and accurate understanding of body image. Further in-depth research on body image perception that incorporates various factors should be conducted.

This study confirmed a significant relationship between BMI, subjective health perception, and body image perception. However, other influential factors such as genetic predisposition, physical activity, and sleep patterns were not included, making it challenging to comprehensively analyze the relationship between BMI and adolescent health issues. Therefore, future studies should incorporate factors such as physical activity, lifestyle habits, and sleep, which influence adolescent health, to conduct a more detailed analysis of the causes of increased BMI. This approach is essential for identifying effective strategies for promoting adolescent health.

## 5. Conclusions

This study analyzed the relationships between BMI, dietary habits, subjective health, and body image perception among Korean adolescents. The general characteristics of Korean adolescents revealed that only 47.1% had a normal BMI, accounting for less than half of the population. Notably, the proportion of overweight adolescents was 29.1%, a sharp increase of 10.1% compared to the previous year, highlighting the alarming rise in obesity rates among Korean adolescents. In terms of dietary habits, the general findings showed a high frequency of water intake and breakfast consumption, along with a low frequency of late-night snacking. However, when examining the dietary habits of high school students specifically, the frequency of water intake was lower, whereas the consumption of soft drinks and late-night snacks was higher. Additionally, this group exhibited higher BMI levels and more negative subjective health and body image perceptions. Furthermore, while male students generally had a higher BMI than female students, subjective health and body image perceptions were higher among female students. However, a correlation was identified between BMI and subjective health and body image perception; although not strong, subjective health was found to have a significant impact on body image perception.

An intriguing finding of this study was that higher water intake, soft drink consumption, and late-night snacking were associated with lower BMI. This result differs from the dietary habits observed among high school students in this study and contrasts with previous research findings. Since factors influencing BMI extend beyond dietary habits, it is essential to conduct a broader investigation into additional contributors to the rising obesity rates among Korean adolescents. A comprehensive and detailed analysis of these factors is required to better understand this issue. BMI not only affects adolescents’ current health but can also influence their health and socioeconomic status as adults. Therefore, it is essential to conduct accurate analyses of the causes, implement preventive policies, and establish strategies to help Korean adolescents maintain healthy BMI and dietary habits while fostering a positive and sustainable perception of health.

As the environment in which adolescents grow and live continues to change, it is crucial to adopt a multifaceted approach to accurately analyze its impact on adolescent health. Comprehensive policies and measures that consider both the mental and physical health of adolescents are expected to significantly improve their health behaviors.

## Figures and Tables

**Table 1:** General characteristics, BMI, dietary habit, subjective cognition of health, and body type of sample.

Group	Participants (*n* = 50,427)		Group	Participants (*n* = 50,427)	
	Variables	*N* (%)		Variables	*N* (%)
Sex	Male	25,731 (51.0)	Breakfast intake (per week)	0 days	11,167 (22.1)
	Female	24,696 (49.0)		1–2 days	8569 (17.0)
School type	Mixed	34,551 (68.5)		3–4 days	7440 (14.8)
	Boys’	7963 (15.8)		5–6 days	9725 (19.3)
	Girls’	7913 (15.7)		7 days	13,526 (26.8)
Grade	7th grade	9027 (17.9)	Late-night snack intake (per week)	0 days	20,352 (40.4)
	8th grade	9094 (18.0)		1–2 days	18,477 (36.6)
	9th grade	9168 (18.2)		3–4 days	8285 (16.4)
	10th grade	8231 (16.3)		5–6 days	2315 (4.6)
	11th grade	7765 (15.4)		7 days	998 (2.0)
	12th grade	7142 (14.2)	Fruit intake (per week)	0 times	5507 (10.9)
BMI (kg/cm^2^)	Underweight	11,958 (23.7)		1–2 times	16,244 (32.2)
	Normal	23,773 (47.1)		3–4 times	14,807 (29.4)
	Overweight/obesity	14,696 (29.1)		5–6 times	5362 (10.6)
				7 or more times	8507 (16.9)
Water intake (per day)	Less than 1 cup	2181 (4.3)	Vegetable intake (per week)	0 times	2132 (4.2)
	1–2 cups	9636 (19.1)		1–2 times	9474 (18.8)
	3 cups	11,329 (22.5)		3–4 times	13,815 (27.4)
	4 cups	8727 (17.3)		5–6 times	7464 (14.8)
	5 or more cups	18,554 (36.8)		7 or more times	17,542 (34.8)
Milk intake (per week)	0 times	8609 (17.1)	Self-related health	be good health	10,308 (20.4)
	1–2 times	14,683 (29.1)		be on the healthy side	21,903 (43.4)
	3–4 times	11,878 (23.6)		be the normality	13,137 (26.1)
	5–6 times	5970 (11.8)		be in bad shape	4764 (9.4)
	7 or more times	9287 (18.4)		to be very unhealthy	315 (0.6)
Soft drink intake (per week)	0 times	3297 (6.5)	Body image perception	be very thin	2485 (4.9)
	1~2 times	15,199 (30.1)		I am a bit skinny	11,016 (21.8)
	3~4 times	17,222 (34.2)		be the normality	18,397 (36.5)
	5~6 times	7278 (14.4)		I have gained some weight	15,540 (30.8)
	7 or more times	7431 (14.7)		I have gained a lot	2989 (5.9)

Note: BMI—body mass index.

**Table 2:** BMI, dietary habits, subjective cognition of health, and body type according to general characteristics.

Group	Variable	BMI	WI	MI	SDI	BI	LNSI	FI	VI	SRH	BIP
		(kg/cm^2^)	(Per Day)	(Per Week)							
Sex	Male	22.20 ± 4.04	3.95 ± 1.16	3.21 ± 1.62	3.20 ± 1.35	4.82 ± 2.76	2.54 ± 1.74	2.99 ± 1.466	3.99 ± 1.68	2.15 ± 0.92	3.06 ± 1.04
	Female	20.53 ± 4.08	3.30 ± 1.29	2.69 ± 1.41	2.97 ± 1.27	4.54 ± 2.67	2.39 ± 1.62	3.00 ± 1.40	3.73 ± 1.64	2.38 ± 0.88	3.16 ± 0.90
	t	51.282 ***	58.810 ***	38.577 ***	19.638 ***	11.419 ***	9.622 ***	−0.730	17.428 ***	−29.349 ***	−11.765 ***
School Type	Mixed (A)	21.17 ± 3.71	3.64 ± 1.27	2.96 ± 1.55	3.08 ± 1.32	4.68 ± 2.72	2.43 ± 1.67	3.00 ± 1.44	3.87 ± 1.67	2.26 ± 0.91	3.08 ± 0.98
	Boys’ (B)	22.74 ± 4.08	3.95 ± 1.15	3.23 ± 1.59	3.21 ± 1.35	4.92 ± 2.75	2.62 ± 1.78	3.00 ± 1.44	4.02 ± 1.67	2.15 ± 0.92	3.12 ± 1.03
	Girls’ (C)	20.95 ± 3.32	3.27 ± 1.30	2.64 ± 1.40	2.99 ± 1.28	2.67 ± 2.67	2.45 ± 1.63	2.96 ± 1.37	3.69 ± 1.64	2.40 ± 0.89	3.23 ± 0.89
	*F*	644.418 ***	589.266 ***	295.514 ***	57.781 ***	60.704 ***	37.888 ***	3.613 *	80.934 ***	163.203 ***	82.874 ***
	Post hoc	B > A > C	B > A > C	B > A > C	B > A > C	B > A > C	B > AC	A > C	B > A > C	C > A > B	C > B > A
Grade	7th (A)	20.42 ± 3.60	3.75 ± 1.23	3.16 ± 1.61	2.95 ± 1.27	4.97 ± 2.71	2.13 ± 1.49	3.18 ± 1.51	3.97 ± 1.72	2.14 ± 0.84	3.08 ± 0.99
	8th (B)	20.78 ± 3.61	3.72 ± 1.24	3.15 ± 1.61	3.07 ± 1.32	4.76 ± 2.73	2.31 ± 1.62	3.13 ± 1.50	3.94 ± 1.70	2.22 ± 0.89	3.07 ± 0.97
	9th (C)	21.22 ± 3.68	3.66 ± 1.26	3.04 ± 1.56	3.06 ± 1.30	4.68 ± 2.74	2.44 ± 1.68	3.05 ± 1.46	3.91 ± 1.68	2.26 ± 0.91	3.08 ± 0.97
	10th (D)	21.72 ± 3.70	3.51 ± 1.30	2.83 ± 1.44	3.17 ± 1.32	4.57 ± 2.70	2.60 ± 1.71	2.90 ± 1.35	3.75 ± 1.62	2.31 ± 0.92	3.12 ± 0.97
	11th (E)	22.08 ± 3.78	3.57 ± 1.29	2.76 ± 1.48	3.15 ± 1.33	4.53 ± 2.71	2.68 ± 1.77	2.83 ± 1.34	3.81 ± 1.63	2.32 ± 0.94	3.13 ± 0.97
	12th (F)	22.43 ± 3.86	3.56 ± 1.29	2.66 ± 1.42	3.17 ± 1.35	4.51 ± 2.70	2.74 ± 1.79	2.81 ± 1.35	3.77 ± 1.64	2.36 ± 0.97	3.20 ± 0.97
	*F*	358.636 ***	49.124 ***	158.613 ***	36.691 ***	34.664 ***	164.132 ***	99.684 ***	24.959 ***	63.836 ***	19.057 ***
	Post hoc	F > E > D > C > B > A	B > DEF, A > C > DEF	AB > C > DE > F	DEF > BC > A	ABC > EF, AB > D	F > DE > C > B > A	AB > C > D > EF	ABC > DEF	DEF > BC > A	F > A, F > E > BC, F > D > B

Note: WI—water intake; MI—milk intake; SDI—soft drink intake; BI—breakfast intake; LNSI—late-night snack intake; FI—fruit intake; VI—vegetable intake; SRH—self-related health; BIP—body image perception; * *p* < 0.05; *** *p* < 0.001.

**Table 3:** BMI according to dietary habits.

Variables	Per Day	BMI (kg/cm^2^)	*F*	Post Hoc
WI	Less than 1 cup (A)	19.93 ± 3.22	531.259 ***	E > D > C > B > A
	1–2 cups (B)	20.43 ± 3.32		
	3 cups (C)	21.01 ± 3.59		
	4 cups (D)	21.45 ± 3.67		
	5 or more cups (E)	22.25 ± 3.97		
MI	0 times (A)	21.22 ± 3.78	14.781 ***	CDE > AB
	1–2 times (B)	21.27 ± 3.68		
	3–4 times (C)	21.45 ± 3.78		
	5–6 times (D)	21.46 ± 3.77		
	7 or more times (E)	21.57 ± 3.84		
SDI	0 times (A)	21.48 ± 3.88	15.362 ***	ABCD > E
	1–2 times (B)	21.41 ± 3.73		
	3–4 times (C)	21.49 ± 3.75		
	5–6 times (D)	21.34 ± 3.77		
	7 or more times (E)	21.09 ± 3.78		
BI	0 days (A)	21.54 ± 3.76	18.666 ***	ABC > D > E
	1–2 days (B)	21.48 ± 3.73		
	3–4 days (C)	21.49 ± 3.77		
	5–6 days (D)	21.33 ± 3.74		
	7 days (E)	21.17 ± 3.80		
LNSI	0 days (A)	21.67 ± 3.87	117.545 ***	A > B > C > DE
	1–2 days (B)	21.44 ± 3.73		
	3–4 days (C)	20.91 ± 3.57		
	5–6 days (D)	20.49 ± 3.55		
	7 days (E)	20.31 ± 3.36		
FI	0 times (A)	21.75 ± 3.92	72.881 ***	AB > C > D > E
	1–2 times (B)	21.59 ± 3.79		
	3–4 times (C)	21.41 ± 3.76		
	5–6 times (D)	21.12 ± 3.64		
	7 or more times (E)	20.87 ± 3.64		
VI	0 times (A)	20.97 ± 3.84	30.638 ***	CDE > ABD > C
	1–2 times (B)	21.09 ± 3.60		
	3–4 times (C)	21.39 ± 3.73		
	5–6 times (D)	21.60 ± 3.79		
	7 or more times (E)	21.49 ± 3.84		

Note: WI—water intake; MI—milk intake; SDI—soft drink intake; BI—breakfast intake; LNSI—late-night snack intake; FI—fruit intake; VI—vegetable intake; *** *p* < 0.001.

**Table 4:** Self-rated health and body type according to BMI.

BMI (kg/cm^2^)	Self-Rated Health			Body Image Perception		
	M ± SD	*F*	Post Hoc	M ± SD	*F*	Post Hoc
Underweight (A)	2.32 ± 0.91	160.704 ***	AC > B	2.11 ± 0.72	26,525.797 ***	C > B > A
Normal (B)	2.19 ± 0.89			3.04 ± 0.70		
Overweight (C)	2.34 ± 0.94			4.03 ± 0.61		

Note: *** *p* < 0.001.

## Data Availability

No new data were created or analyzed in this study.
